# miR-135a Regulates Atrial Fibrillation by Targeting Smad3

**DOI:** 10.1155/2023/8811996

**Published:** 2023-05-05

**Authors:** Xueting Fan, Kai Feng, Yonghui Liu, Leixi Yang, Yizhuo Zhao, Liping Tian, Yiqun Tang, Xiaozhi Wang

**Affiliations:** ^1^Department of Clinical Pharmacy, School of Basic Medicine and Clinical Pharmacy, China Pharmaceutical University, Nanjing 211198, China; ^2^Department of Pharmacy, The First Affiliated Hospital with Nanjing Medical University, Nanjing 210029, China; ^3^Department of Pharmacy, Ningbo First Hospital, Ningbo Hospital of Zhejiang University, Ningbo 315010, China; ^4^Department of Cardiology, The First Affiliated Hospital with Nanjing Medical University, Nanjing 210029, China

## Abstract

**Background:**

Atrial fibrillation (AF) is the most common arrhythmia in clinical. Atrial fibrosis is a hallmark feature of atrial structural remodeling in AF, which is regulated by the TGF-*β*1/Smad3 pathway. Recent studies have implicated that miRNAs are involved in the process of AF. However, the regulatory mechanisms of miRNAs remain largely unknown. This study is aimed at investigating the function and regulatory network of miR-135a in AF.

**Methods:**

*In vivo*, the plasma was collected from patients with AF and non-AF subjects. Adult SD rats were induced by acetylcholine (ACh) (66 *μ*g/ml)-CaCl_2_ (10 mg/ml) to establish an AF rat model. *In vitro*, atrial fibroblasts (AFs), isolated from adult SD rats, were treated with high-frequency electrical stimulation (HES) (12 h) and hypoxia (24 h) to mimic the AF and atrial fibrosis, respectively. miR-135a expression was detected through quantitative real-time polymerase chain reaction (qRT-PCR). The association between miR-135a and Smad3 was speculated by the TargetScan database and confirmed by the luciferase reporter assay. Fibrosis-related genes, Smad3, and TRPM7 were all assessed.

**Results:**

The expression of miR-135a was markedly decreased in the plasma of AF patients and AF rats, which was consistent with that in HES-treated and hypoxia-treated AFs. Smad3 was identified as a target of miR-135a. the downregulation of miR-135a was associated with the enhancement of Smad3/TRPM7 expressions in AFs. Additionally, the knockdown of Smad3 significantly reduced the expression of TRPM7 and further inhibited atrial fibrosis.

**Conclusions:**

Our study demonstrates that miR-135a regulates AF via Smad3/TRPM7, which is a potential therapeutic target for AF.

## 1. Introduction

Atrial fibrillation (AF), the most common sustained arrhythmia encountered in humans, is an important contributor to morbidity and mortality. Atrial fibrosis plays a key role in the development and persistence of AF because fibrotic remodeling promotes arrhythmogenesis through impaired conduction and subsequent generation of reentry circuits [1, 2]. Atrial fibrosis acts as both a trigger and a by-product of AF. Transforming growth factor-*β*1 (TGF-*β*1)/Smads pathway is considered to be the canonical pathway leading to fibrosis in various tissues, especially in atrial fibrosis [3]. TGF-*β*1 activates Smad2 and Smad3 by the TGF-*β* receptor 1 (TGFR1), and then Smad4 binds activated Smad2/3. This complex translocates to the nucleus. The Smad3 component of the complex binds directly to gene promoters to induce transcription of profibrotic molecules, including collagen I and *α*-smooth muscle actin (*α*-SMA), which induce myofibroblast activation and matrix deposition [4].

MicroRNAs (miRNAs), noncoding RNA molecules, are approximately 22 nt in length and participate in the regulation of posttranscriptional gene expression. Mature miRNAs bind to the 3' untranslated region (3′-UTR) of mRNA complementarily and restrict translation or induce degradation of target mRNA [5]. Several studies have shown that miRNAs participate in cardiovascular diseases, such as heart failure, AF, and ischaemic heart disease [6, 7]. miRNAs are intimately associated with cardiac fibrosis. For example, miR-451a exhibits antifibrotic properties [8], while miR-21 exerts promote fibrotic effect [9]. Previous research has found that miR-135a expression is downregulated in ISO-induced cardiac fibrosis [10]. Emerging researches have confirmed that miRNAs are closely related with Smad3. For instance, miR-671-5p can inhibit the invasion and migration of osteosarcoma cells by negatively regulating Smad3 [6]. Based on the TargetScan database, Smad3 was predicted to be a potential target of miR-135a. However, the function of the miR-135a in AF remains unclear, which requires further exploration.

Transient receptor potential melastatin 7 (TRPM7) possesses ion channel and protein kinase functions [11, 12]. It is involved in the pathogenesis of fibrotic diseases including AF and is elevated in patients with AF [13, 14]. We have previously determined that TRPM7 is one target of miR-135a by luciferase reporter assay. Moreover, there is positive feedback between TGF-*β*1 and TRPM7, both of which can promote the development of myocardial fibrosis [15].

In this study, the investigation of miR-135a in AF patients and AF models showed that the expression of miR-135a was significantly reduced in AF. The downregulation of miR-135a was associated with atrial fibrosis. We further identified miR-135a inhibited atrial fibrosis by regulating Smad3/TRPM7. All of the above results suggest miR-135a may provide a new potential treatment for AF.

## 2. Materials and Methods

### 2.1. Human Plasma Samples

In the present study, patients in the AF group (*n* = 9) were hospitalized in The First Affiliated Hospital with Nanjing Medical University from August 2017 to September 2017 with a diagnosis of any type of AF; patients in the control group (*n* = 5) were hospitalized during the same period without the diagnosis of AF, and the baseline data were shown in Table 1. Plasma samples were stored at −80°C.

### 2.2. Animals

Adult male SD rats (weight 200–220 g) were purchased from the Qinglongshan animal breeding farm. Rats were randomly divided into three groups (*n* = 10 per group) as follows: control group, AF group, and amiodarone treatment group. The rats in AF and amiodarone treatment group were injected with acetylcholine (ACh) (66 *μ*g/ml)-CaCl_2_ (10 mg/ml) mixture (Sigma-Aldrich, USA) through tail vein (0.1 ml/100 g/day) for 10 days to establish the AF model. The appearance of a typical AF electrocardiogram (ECG) is considered a successful model of AF. Meanwhile, the rats in the amiodarone treatment group were intraperitoneally injected with amiodarone (3 mg/100 g/day) (Sanofi, France) on day 4. The control group was injected with an equal volume of saline. On day 11, plasma was collected from three groups, all rats were euthanized, and atria were harvested for further analysis.

### 2.3. Histological Analysis

Atrial tissues were obtained from the three rat groups, and the tissues were fixed and embedded in paraffin. Small sections (5 *μ*m) of the fixed embedded tissues were cut. Tissue sections were stained with hematoxylin-eosin staining (HE) for morphological evaluation and Masson's trichrome stain for assessing the degree of fibrosis. The collagen volume fraction was determined by calculating the collagen area/total area using ImageJ software.

### 2.4. Electrophysiological Investigation

ECG, atrial effective refractory period (AERP), and the duration of AF were measured as previously described in three groups of rats [16]. ECG was recorded immediately after the ACh-CaCl_2_ mixture injection. In brief, the P wave disappears and the F wave appears in ECG, indicating that AF begins while the end is designated by the appearance of normal ECG. The interval time is the duration of AF. AERP was measured by the BL-420 system of Tai Meng (Cheng Du) using an incremental technique, with 1 ms steps at basic drive cycle lengths of 50 ms.

### 2.5. Isolation and Treatment of AFs

Primary atrial fibroblasts (AFs) were isolated from normal adult SD rats as Fajin et al. described [17]. Briefly, SD rats were euthanized, and the hearts were rapidly excised in a sterile environment; atria were isolated from the hearts and cut into 1 mm^3^ sections. The sections were then placed in a culture flask and incubated in a 5% CO_2_ incubator at 37°C. Next, Dulbecco's modified eagle's medium (DMEM) containing 15% fetal bovine serum (FBS), 100 U/ml penicillin, and 100 *μ*g/ml streptomycin was added after 4 hours. The culture medium was changed every 2 days, and confluent cells at passage numbers 2-3 were used for the experiments. AFs were treated with high-frequency electrical stimulation (HES) and hypoxia to establish the AF and atrial fibrosis model *in vitro*, respectively. For HES, AFs were seeded in 6-well plates and treated with C-Pace100TM Cell electrical stimulator (IonOptix, Netherlands). The stimulation parameter was set based on Jiang et al. [18]. The parameter is as follows: 5 ms duration, 17 Hz square-wave pulses, and 7 V/cm. For hypoxia, the cells were incubated in the three-gas incubator (93% N_2_, 5% CO_2_, and 2% O_2_) for 0 h, 6 h, 12 h, and 24 h. Cells and cellular supernatants were collected for further study.

### 2.6. Small Interfering RNA Transfection

Small interfering RNA (siRNA) oligonucleotides against Smad3 genes or scrambled sequences, which served as a negative control (NC), were synthesized by GenePharma (Shanghai, China). The sequences were the following: Smad3-siRNA (sense: 5′-UGGUGCGAGAAGGCGGUCATT-3′; antisense: 5′-UGACCGCCUUCUCGCAC CATT-3′); NC (sense: 5′-UUCUCCGAACGUGUC ACGUTT-3′; antisense: 5′-ACGUGACACGUUCGGAGAATT-3′).

### 2.7. Quantitative Real-Time Polymerase Chain Reaction (qRT-PCR)

Total RNA of atrial tissues and AFs were extracted by Trizol reagent (Beyotime, China). cDNA was synthesized with HiScript II Q RT SuperMix for qPCR kit, and the mRNA level was quantified by ChamQ SYBR qPCR Master Mix kit (Vazyme Biotech, China). miRNA of plasma and cellular supernatant was extracted by miRNeasy Serum/Plasma Kit (QIAGEN, Germany), and miR-135a first-strand cDNA was synthesized with miRNA 1st Strand cDNA Synthesis Kit (by stem-loop) (Vazyme Biotech, China). The level of miR-135a was measured by miRNA Universal SYBR qPCR Master Mix (Vazyme Biotech, China). U6 and cel-miR-39 were used as the standard control to normalize the expression of miR-135a. All sequences of primers were listed in Table 2. The expression of mRNA and miR-135a was determined by the formula: 2^−△△Ct^.

### 2.8. Western Blotting

Western blotting was performed according to standard methods. Bands in the developed images were quantified using ImageJ. The primary antibodies used were TRPM7 (Boster, China), Smad3 (CST, USA), *α*-smooth muscle actin (*α*-SMA) (Abclonal, China), Collagen I (Bioss, China), and GAPDH (Proteintech, China). The relative protein expressions were normalized to GAPDH values.

### 2.9. Luciferase Reporter Assay

HEK-293 cells (originally purchased from ATCC) were seeded into 24-well plates and after 24 h incubation, the confluence reached 60-70%. Then, the pmirGLO luciferase constructs containing the wild-type or mutants of 3′-UTR fragments of Smad3 were cotransfected with miR-135a mimics or inhibitors into HEK-293 cells using Lipofectamine 3000 (Invitrogen, USA). At 24 h after cotransfection, firefly and renilla luciferase activities were assayed using a dual-luciferase reporter assay system (Promega, USA) according to the manufacturer's instructions. The firefly luciferase activity was normalized by renilla luciferase activity.

### 2.10. Statistical Analysis

Independent experiments were performed at least three times (*n* ≥ 3). All data were represented as mean ± standard deviation (SD), Student's *t*-test was used to analyze data between two groups, and multiple-group comparisons were performed by one-way ANOVA. The data were analyzed by GraphPad Prism 7.0, and *P* < 0.05 was considered statistically significant.

## 3. Results

### 3.1. miR-135a Is Downregulated in AF Patients and Rats

**Figure 1:**
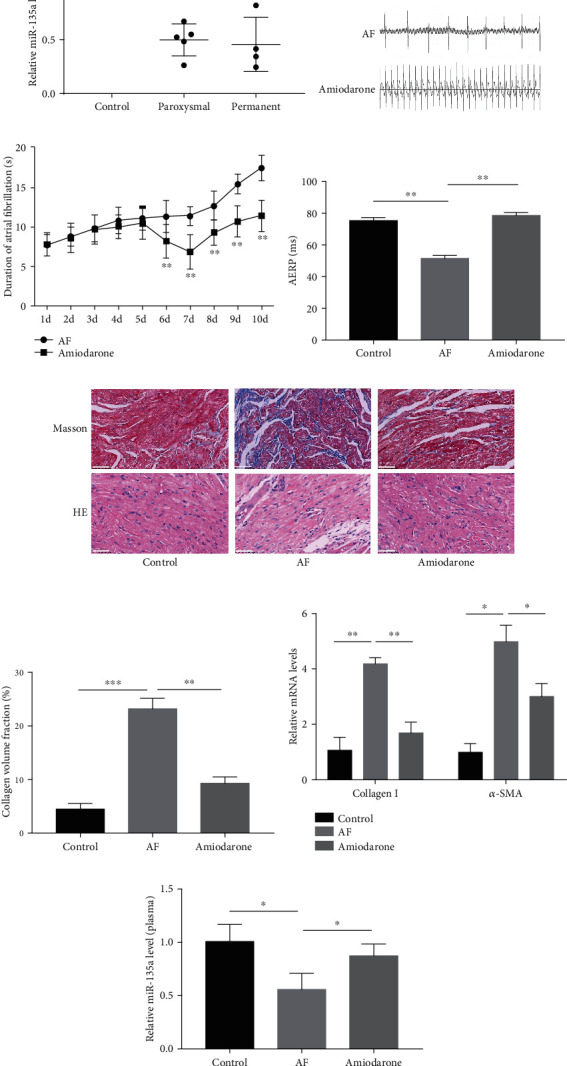
miR-135a was downregulated in AF patients and rats. (a) Relative expressions of miR-135a in the plasma of humans were detected by qRT-PCR; ECG (b), the duration of AF (c), and AERP (d) of rats in the control, AF, and amiodarone treatment group; representative Masson stain and HE stain (e) and collagen volume fraction (f) of rat atrial tissues in the control, AF, and amiodarone treatment group (in Masson stain group: collagen fiber (blue), muscle tissue (red); in HE stain group: nucleus (blue), cytoplasm (red); scale bar = 50 *μ*m); (g) mRNA levels of collagen I and *α*-SMA in atrial tissues in each group; (h) relative expression of miR-135a in plasma of rats in the control, AF, and amiodarone treatment group. (^∗^*P* < 0.05, ^∗∗^*P* < 0.01, ^∗∗∗^*P* < 0.001).

To investigate whether miR-135a is involved in AF, we examined the expression of miR-135a in the plasma of AF patients. As shown in Figure 1(a), the level of miR-135a expression in paroxysmal and permanent AF patients was both significantly lower than that in the control group. There was no significant difference between paroxysmal and permanent AF patients. It suggested that miR-135a was downregulated in the plasma of patients with AF.

To further explore the potential association between miR-135a and AF, SD rats were injected with ACh-CaCl_2_ to establish an AF model. A typical rat AF ECG appeared after ACh-CaCl_2_ treatment (Figure 1(b)), which indicated that the AF model was successfully established. Meanwhile, the duration of AF increased and AERP decreased in AF rats (Figures 1(c) and 1(d)). AERP returned to normal level after amiodarone treatment (as a positive control) (Figure 1(d)). We performed the Masson and HE staining on the atria of rats, and found that atrial fibrosis was indeed aggravated in AF rats and attenuated after amiodarone treatment (Figure 1(e)). Quantitative analysis showed the same trend, and the collagen volume fraction of rats' atrial was markedly increased in the AF group and reduced after amiodarone treatment (Figure 1(f)). The further detection of fibrosis-related gene expression demonstrated that the mRNA levels of collagen I and *α*-SMA were markedly elevated in the atrial tissues of AF rats and downregulated by amiodarone therapy (Figure 1(g)). All of these results further indicated that the model of AF rat was successfully established, and AF was associated with atrial fibrosis.

We detected miR-135a in the plasma of AF rats. As shown in Figure 1(h), the level of miR-135a expression was significantly downregulated in AF rats, which was consistent with that in AF patients, and miR-135a was restored to the normal level in the amiodarone treatment group. We also examined the expression of miR-135a in the atrial tissues of AF rats (Supplementary Figure 1). There was no significant difference between the AF group and the control group. These results suggest that the expression of miR-135a is stably downregulated in the plasma of AF.

### 3.2. miR-135a Is Downregulated in HES and Hypoxia-Treated AFs *In Vitro*

**Figure 2:**
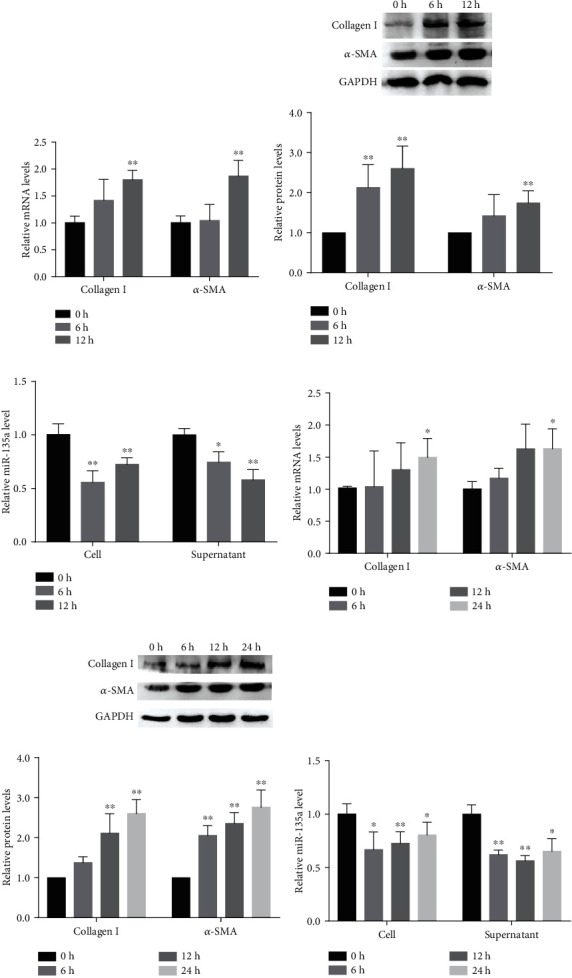
miR-135a was downregulated in HES and hypoxia-treated AFs. mRNA (a) and protein (b) levels of collagen I and *α*-SMA in AFs treated with HES for 0 h, 6 h, and 12 h. (c) Relative expression of miR-135a in HES-stimulated AFs and cellular supernatants; mRNA (d) and protein (e) levels of collagen I and *α*-SMA in AFs after hypoxia 0 h, 6 h, 12 h, and 24 h. (f) Relative expression of miR-135a in hypoxia-stimulated AFs and cellular supernatants. The levels of proteins were relative to GAPDH expression. (^∗^*P* < 0.05, ^∗∗^*P* < 0.01).

Rapid stimulation can lead to electrical remodeling of the atria, which is an important mechanism for the occurrence and persistence of AF [19]. AFs isolated from normal SD rats were treated with HES for 0 h, 6 h, and 12 h to mimic AF *in vitro*. The expression of fibrosis-related genes collagen I/*α*-SMA was notably increased after 12 h of HES (Figures 2(a) and 2(b)). Finally, HES for 12 h was selected to mimic AF *in vitro*. We further found that the level of miR-135a expression in both AFs and cellular supernatant was decreased after HES treatment (Figure 2(c)), consistent with the trend of miR-135a expression *in vivo*.

Hypoxia is reported as the basic pathogenesis in several cardiovascular diseases, including AF [20]. In response to hypoxia, fibroblasts proliferate and differentiate, which aggravates the degree of fibrosis. In our current study, AFs were treated with hypoxia to establish an atrial fibrosis model *in vitro.* As shown in Figures 2(d) and 2(e), the expression levels of collagen I and *α*-SMA were significantly elevated after hypoxia treatment for 24 h, which indicated that the model of atrial fibrosis was successfully established after hypoxia for 24 h in AFs. As expected, the level of miR-135a in both AFs and cellular supernatants was also downregulated after hypoxia stimulation (Figure 2(f)). Collectively, these data reveal that the expression of miR-135a is stably downregulated in AF.

### 3.3. Smad3 Is a Direct Target of miR-135a

**Figure 3:**
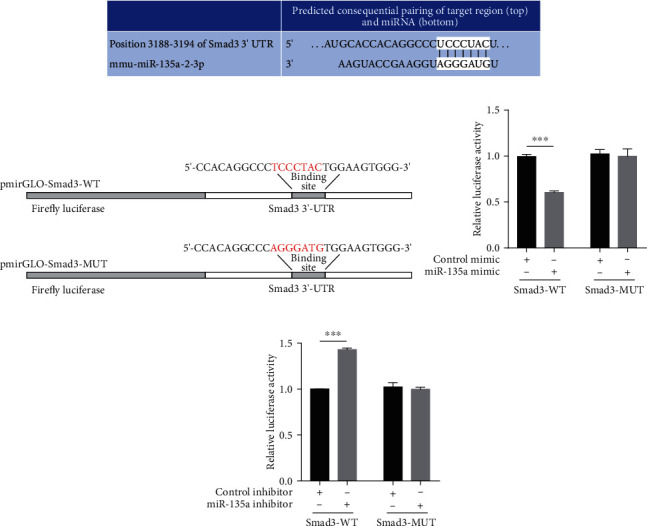
miR-135a directly regulated Smad3 expression. (a) TargetScan database predicted interactions between miR-135a and their binding site in the Smad3 3′-UTR; (b) fragments of the Smad3 3′-UTR were cloned into pmirGLO reporter plasmids. The mutated nucleotides are marked in red. HEK293 cells were transfected with miR-135a mimics (c) or inhibitors (d) along with the indicated reporter plasmids for 24 h. The relative luciferase activities were analyzed. (^∗∗∗^*P* < 0.001).

To further investigate the regulatory mechanism of miR-135a in AF, the potential targets of miR-135a were predicted by the TargetScan database. As shown in Figure 3(a), the 3′-UTR of Smad3 contains a conserved miR-135a binding site. Due to the regulation of Smad3 which is possibly mediated by the binding of miR-135a to the predicted sites in the Smad3 3′-UTR, we cloned the Smad3 3′-UTR harboring the wild type (Smad3-WT) or mutant (Smad3-MUT) miR-135a target sequence into pmirGLO plasmids (Figure 3(b)). The Smad3-WT or Smad3-MUT reporter plasmids were transfected into HEK293 cells along with miR-135a mimic, inhibitor, or the corresponding negative control RNAs. Overexpression of miR-135a suppressed luciferase reporter activity (Figure 3(c)), whereas inhibition of miR-135a resulted in the opposite effect (Figure 3(d)). By contrast, neither overexpression nor inhibition of miR-135a affected the luciferase activity of the mutant reporter (Figures 3(c) and 3(d)). Taken together, these results show that miR-135a directly regulates Smad3 expression by targeting the 3′-UTR of Smad3.

### 3.4. miR-135a Is Involved in AF by Negatively Regulating Smad3/TRPM7

**Figure 4:**
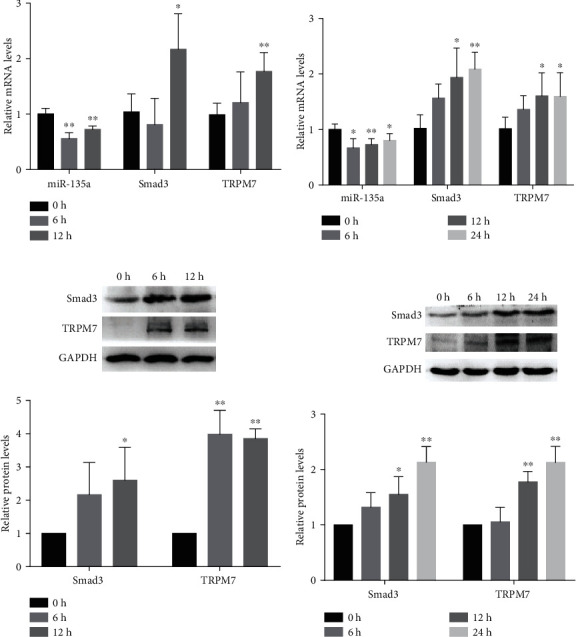
Smad3 and TRPM7 were upregulated in AF and showed a negative correlation with miR-135a. The expression of miR-135a and mRNA levels of Smad3 and TRPM7 in AFs induced by HES (a) and hypoxia (b); Relative protein levels of Smad3 and TRPM7 in AFs treated with HES (c) and hypoxia (d). The levels of proteins were relative to GAPDH expression. (^∗^*P* < 0.05, ^∗∗^*P* < 0.01).

In order to assess the correlation of Smad3 with miR-135a in AF, we examined the expression level in HES-stimulated AFs. The results showed that Smad3 was significantly upregulated at mRNA levels, accompanied by the deceased expression of miR-135a, specially treated with HES for 12 h (Figure 4(a)). In addition, the same trend was observed in hypoxia-induced AFs (Figure 4(b)). Noticeably, all the results confirmed the negative correlation between miR-135a and Smad3.

Since TRPM7 has been proven to be involved in the fibrogenesis of AF [13], and TRPM7 is one target of miR-135a [15], we hypothesized that the underlying mechanism by which miR-135a regulates AF is related to TRPM7. The expression of TRPM7 was consequently examined using qRT-PCR and western blotting in HES and hypoxia-induced AFs. As shown in Figures 4(a)–4(d), the same as Smad3, the level of TRPM7 was obviously upregulated in AFs after 12 h HES and 24 h hypoxia treatment.

**Figure 5:**
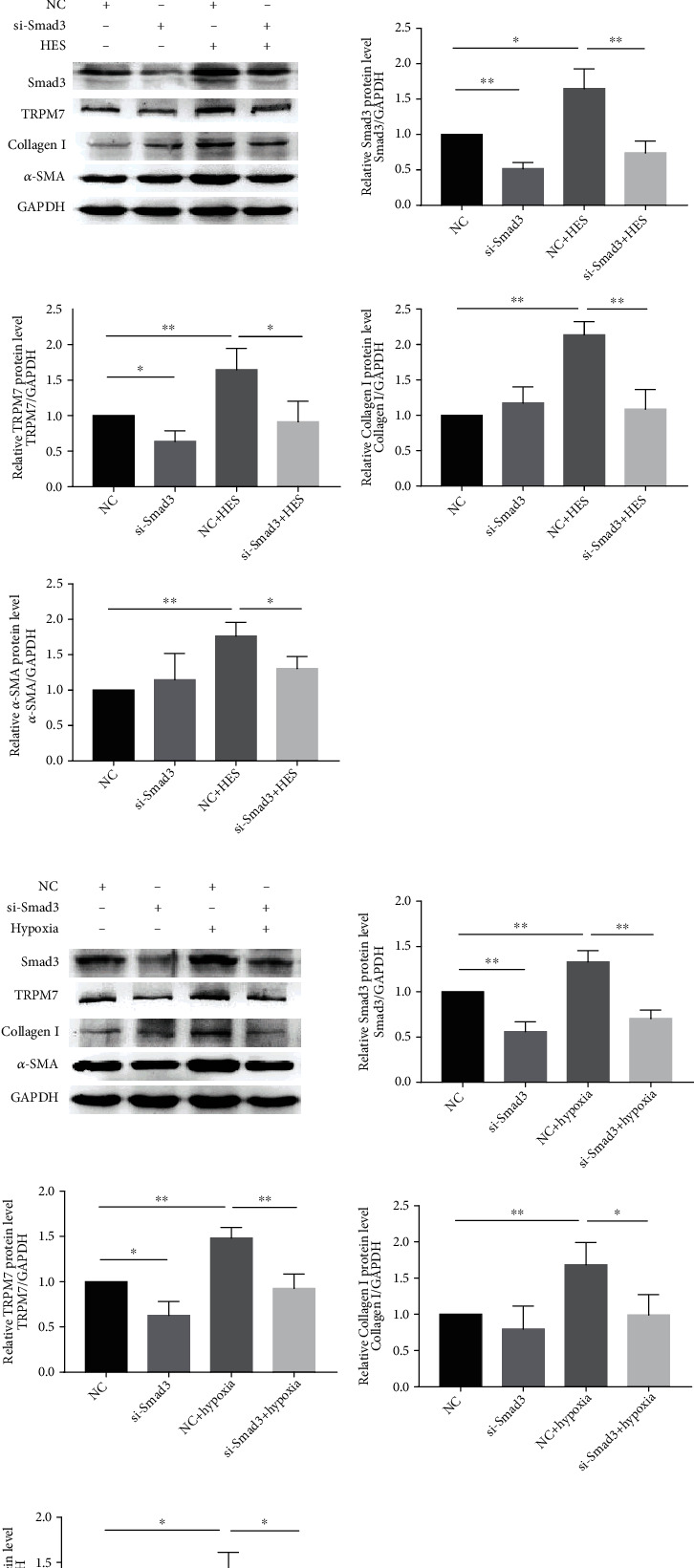
miR-135 regulated AF via Smad3/TRPM7. (a) Knockdown efficiency of two different interference sequences of Smad3 was determined by western blotting; AFs were transfected with Smad3 siRNA and then treated with HES for 12 h (b) or hypoxia for 24 h (c); cells were harvested and subjected to western blotting analysis of Smad3, TRPM7, collagen I, and *α*-SMA protein expression. The levels of proteins were relative to GAPDH expression (^∗^*P* < 0.05, ^∗∗^*P* < 0.01).

A great deal of researches indicates that TGF-*β*1/Smad3 pathway is involved in the fibrosis process of many tissues [21, 22]. Our previous studies have revealed that there is a positive feedback between TGF-*β*1 and TRPM7, both of which are involved in myocardial fibrosis [15]. The above results showed that the expressions of Smad3 and TRPM7 were obviously increased in HES and hypoxia-treated AFs. Subsequently, we designed two Smad3 siRNAs and then detected the efficiency of Smad3 knockdown in AFs after 48 h transfection (Figure 5(a)). siRNA-2 was used in the following studies. The results in Figures 5(b) and 5(c) indicated that Smad3-siRNA remarkably suppressed TRPM7 expression level, especially in long-term HES (12 h) and hypoxia-induced (24 h) AFs. These results confirmed that Smad3 positively regulated the expression of TRPM7 in AFs. What is more, knock-down Smad3 significantly alleviated atrial fibrosis in AFs after HES and hypoxia stimulation (Figures 5(b) and 5(c)). Given that miR-135a can negatively affect Smad3 expression, these data suggest that the downregulation of miR-135a promotes atrial fibrosis through Smad3/TRPM7 in AFs.

## 4. Discussion

Atrial fibrosis is involved in AF and is a characteristic of AF substrate [23, 24]. Many studies have confirmed that the TGF-*β*1/Smad3 signaling pathway plays a leading role in fibrosis [25]. Smad3 pathway is involved in atrial fibrosis in AF [26]. Recent studies have shown that miRNAs can inhibit fibroblast proliferation and pulmonary fibrosis by regulating Smad3 [27, 28].

miRNAs are endogenous, noncoding RNAs that negatively regulate a variety of target genes [5]. Abnormal miRNA expression results in several diseases, including cardiovascular disorders and cancers [29]. Extensive researches have shown that miRNAs exist stably in serum and plasma so that the detection of miRNA in serum or plasma can be used as a diagnostic standard for human diseases [30]. Moreover, many miRNAs have been reported to mediate the regulation of AF [31]. Overexpression of miR-27b-3p attenuates atrial fibrosis in rats with AF via the Wnt/*β*-Catenin signaling pathway [32]. Inhibition of miR-455-5p expression effectively ameliorated AF by targeting the suppressor of cytokines signaling 3 [33]. In this study, we found the expression of miR-135a significantly decreased in the plasma of patients with AF compared with people with normal sinus rhythm. Besides, the miR-135a expression level in the plasma of AF rats was consistent with it in AF patients. In the amiodarone-treated group, the expression of miR-135a was restored to the normal level, indicating that miR-135a was downregulated in AF. Furthermore, we examined the expression of miR-135a in the atrial tissues of the AF rat model, as shown in Supplementary Figure 1. We did not find an obvious difference in miR-135a level between the AF group and the control group, which was inconsistent with the results in the plasma of AF models. We believe that the difference between these two results is due to the different levels of miR-135a in various cellular components of atrial tissue, including atrial fibroblasts, atrial cardiomyocytes, endothelial cells, pericytes, and smooth muscle cells [34], as well as the limitation of sample size. The specific reasons leading to this result should be explored in future research.

Emerging evidence suggests that miR-135a participates in the processes of cardiovascular disease and plays a protective role. For instance, miR-135a protects against myocardial ischemia-reperfusion injury by targeting protein tyrosine phosphatase 1B [35], and Feng et al. reported that miR-135a ameliorated ISO-induced cardiac injury by targeting the TLR4 [36]. Our results revealed that miR-135a may act as a fibrosis-regulated factor, which was significantly downregulated in both HES and hypoxia-treated AFs and cellular supernatant, accompanied by the aggravation of atrial fibrosis. We further explored the association between miR-135a and Smad3, which revealed that miR-135a negatively regulated Smad3 by targeting the 3′-UTR of Smad3.

TRPM7 is the major Ca^2+^-permeable channel in AFs and is markedly upregulated in patients with AF [13]. Previous research has revealed that TGF-*β*1 increases the expression of TRPM7 in airway smooth muscle cells (ASMC) through the TGF*β*R/Smad3 pathway [37]. Wei et al. and Fang et al. found that there was a positive feedback loop between TRPM7 and TGF-*β*1/Smads signaling in myocardial and hepatic fibrogenesis [15, 38]. In this study, HES and hypoxia stimulation elevated TRPM7 expression in AFs. Meanwhile, Smad3 siRNA decreased the expression of TRPM7 and inhibited fibrosis in AFs. All of these findings suggested that TGF-*β*1 regulated TRPM7 expression via Smad3 in atrial fibrosis. Previous research in our lab has shown that TRPM7 is one target of miR-135a [15]. Therefore, miR-135a can regulate TRPM7 by targeting Smad3 and directly targeting TRPM7. These findings indicated that miR-135a alleviated atrial fibrosis via Smad3/TRPM7.

In this study, we investigated the mechanism of miR-135a in AF patients and AF rat models *in vivo.* We treated primary AFs with HES to mimic abnormal electrical activity in AF and established an atrial fibrosis model by hypoxia on AFs *in vitro*. Admittedly, the lack of improved evidence of miR-135a in the AF process is a limitation of this study. The expression and the specific loci of miR-135a in atrial tissue should be further explored to clarify the effect of miR-135a in AF. We will further explore the evidence of miR-135a in AF in the future.

## 5. Conclusions

In conclusion, our study showed that miR-135a was stably downregulated in AF both *in vivo* and *in vitro*, which was accompanied by atrial fibrosis. miR-135a directly regulated the expression of Smad3 in AF and it played an antifibrotic role in AF via Smad3/TRPM7 pathway. This study provides new insights into the role of miR-135a in AF and a novel potential therapeutic strategy in AF.

## Figures and Tables

**Table 1:** Baseline data of patients.

	AF (*n* = 9)	SR (*n* = 5)
Median age	66 (45-86)	51 (35-63)
AF types (paroxysmal/permanent)	5/4	—
Male/female	4/5	3/2
Hypertension	4	4
CHD	2	1
Diabetes mellitus	1	2
Surgeries	0	0

SR: sinus rhythm; CHD: coronary heart disease.

**Table 2:** Primer sequences for qRT-PCR.

Gene		5′-3′
TRPM7	F	TGCCATCTGAAGGAGGAACA
	R	ACTCTGCGACAGCCTCATCA
Smad3	F	CTTCACAGCCGTCCATGACAGTAG
	R	CCAATGTAGTAGAGCCGCACACC
*α*-SMA	F	AGGAGCATCCGACCTTGCTA
	R	GCACAGCCTGAATAGCCACA
Collagen I	F	GAGCGGAGAGTACTGGATCGA
	R	CTGACCTGTCTCCATGTTGCA
GAPDH	F	TTTGAGGGTGCAGCGAACTT
	R	ACAGCAACAGGGTGGTGGAC
miR-135a	F	CGCGTATGGCTTTTTATTCCT
	R	AGTGCAGGGTCCGAGGTATT
Cel-miR-39	F	GCGTCACCGGGTGTAAATC
	R	AGTGCAGGGTCCGAGGTATT
U6	F	CTCGCTTCGGCAGCACA
	R	AACGCTTCACGAATTTGCGT
miR-135a-RT		GTCGTATCCAGTGCAGGGTCCGAGGTATTCGCACTGGATACGACTCACAT
Cel-39-RT		GTCGTATCCAGTGCAGGGTCCGAGGTATTCGCACTGGATACGACCAAGCT

## Data Availability

The analyzed data used to support the findings of this study are available from the corresponding author upon request.

## Supplementary Materials

Supplementary Materials Supplementary Figure 1: relative expression of miR-135a in the atrial tissues of rats was detected by qRT-PCR.
