# Supplementary material for: miR-135a Regulates Atrial Fibrillation by Targeting Smad3
Source: Cardiovasc Ther. 2023 May 5;2023:8811996. doi: 10.1155/2023/8811996 (PMC10181910; doi:10.1155/2023/8811996)
Supplement: Supplementary Materials — Supplementary Figure 1: relative expression of miR-135a in the atrial tissues of rats was detected by qRT-PCR. [file 8811996.f1.docx]

**Supplementary materials**

**Supplementary Figure 1:** Relative expression of miR-135a in the atrial tissues of rats was detected by qRT-PCR (n=3; ns, no significance)
